# Supplementary material for: Integration of single photon emitters in 2D layered materials with a silicon nitride photonic chip
Source: Nat Commun. 2019 Sep 30;10:4435. doi: 10.1038/s41467-019-12421-0 (PMC6768863; doi:10.1038/s41467-019-12421-0)
Supplement: Supplementary file 1 — Supplementary Information [file 41467_2019_12421_MOESM1_ESM.pdf]

Supplementary Information:

Integration of Single Photon Emitters in 2D Layered Materials with a Silicon Nitride Photonic Chip

Peyskens et al.

Supplementary Note 1: Fabrication

The silicon nitride waveguides were patterned using standard e-beam lithography on a Raith Voyager system. A positive e-beam resist ARP-6200.09 was spincoated (3000 RPM, baked at 150°C for 1 min) on a commercially grown slab wafer (220 nm SiN on SiO_2 on Si). Subsequently a protective coating (Electra 92) was spincoated (2000 RPM, baked at 90°C for 2 min) on top of the resist. Air trenches (3 μm wide) were defined in FBMS mode (Fixed Beam Moving Stage) to avoid stitching errors at the boundaries of the writing field. The Electra 92 coating can be removed by DI water. After development and etching, a 700 nm wide waveguide remains between the air trenches. The samples were thoroughly cleaned before dry transfer. WSe_2 flakes were mechanically exfoliated from a bulk crystal purchased from hqgraphene. The flakes were subsequently picked up by a GelPak stamp and transferred to the SiN surface by gently releasing the GelPak stamp from the substrate.

Supplementary Note 2: Experimental setup

The setup is shown in Supplementary Figure 1. The cryostat has a top window to excite the sample from above (X symbol highlighting the propagation direction of the excitation beam) and through which the free-space PL is collected ($\circ$ symbol highlighting the propagation direction of the PL beam). A system of two galvo-mirrors allows to scan the excitation beam over the sample through the top window of the cryostat. One of the side ports is equipped with a home-built vacuum fiber feedthrough to minimize transmission losses between the cryostat and the outside world. Depending on the experiment, FM1 can be flipped to excite the sample with variable wavelengths from an Msquared Ti:saph laser. The dichroic mirror (DM) filters the green excitation beam from the PL. The PL collected in the fiber is sent to a fiber collimator unit and is subsequently coupled in the same path used for characterization of the free-space PL by flipping FM2 (this also hinders any free-space PL to be collected while we are studying the waveguide-coupled PL). A longpass filter (LPF) filters out remaining contributions from the pump beam. Without FM3, the PL is sent into a free-space spectrometer. When flipping FM3, the same beam is sent to an HBT setup for second-order correlation measurements. Combination of the half-wave plate with the polarizing beamsplitter allows to balance the counts on the 2 SPDs (the beams are focused by a lens on the surface of the free-space SPDs) and allows to study the relative polarization between the excitation and PL beam.

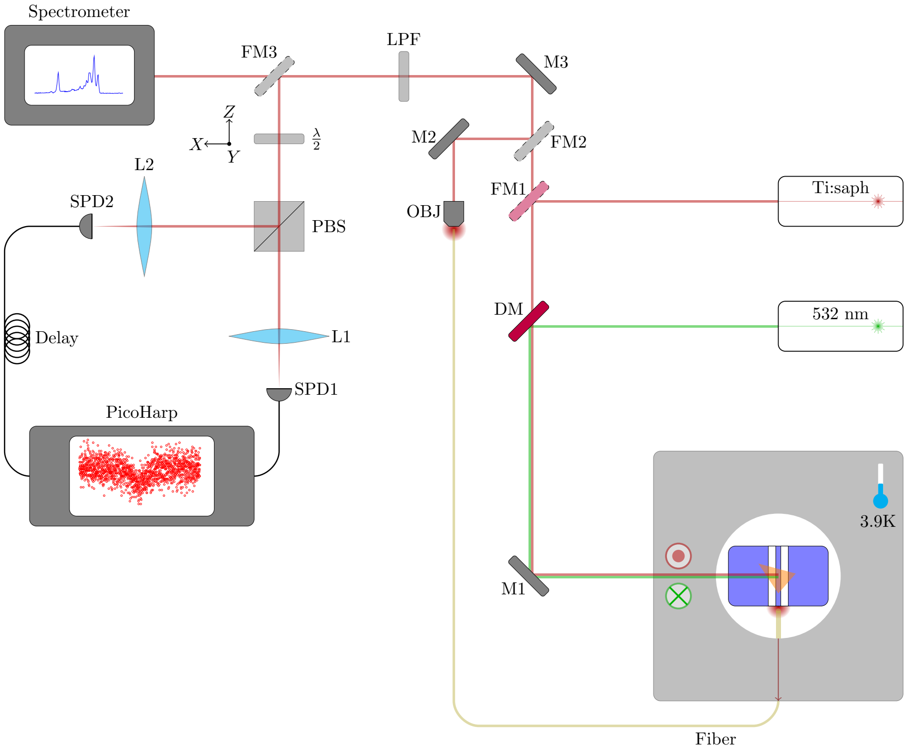

Supplementary Figure 1. Measurement setup. M: fixed mirror, FM: flip mirror, DM: dichroic mirror, OBJ: fiber collimating objective, LPF: longpass filter (either for 532 nm or 715 nm), $\frac{\lambda}{2}$: half-wave plate, PBS: polarizing beamsplitter, L: focusing lens, SPD: Single Photon Detector

Supplementary Note 3: Delocalized exciton defect band spectrum

A typical spectrum of the flake (measured with 532 nm excitation and excitation power of 80 μW) is shown in Supplementary Figure 2(a). One can see the neutral exciton peak around 710 nm (1.75 eV) with the broad delocalized neutral exciton defect band.

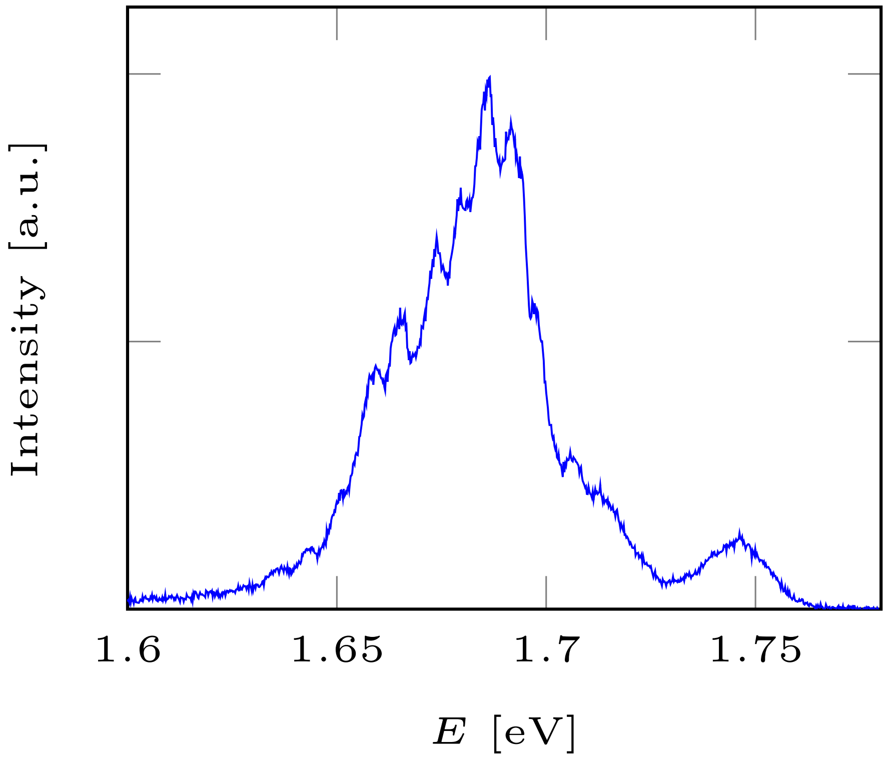

Supplementary Figure 2. Delocalized exciton defect band spectrum.

Supplementary Note 4: Hyperspectral scan of the integrated emitters

This sections contains data from a hyperspectral scan of an area near the waveguide (highlighted by the blue-dashed area in Figure 2(c) of the main text). For each point in the scan we took a spectrum, calculated the total spectral count and normalized the spectral count in different narrower subbands (each 10 nm wide) to this total count (Fig.3(a1-h1)). For each spatial point, we also assessed the number of clear peaks in the different spectral subbands (Fig.3(a2-h2)). One can see that the PL emission predominantly consists of peaks with a wavelength in the 720 nm to 760 nm range and that in each subband of 10 nm, the average number of peaks ranges from 1 to 3, i.e. an average of 4 to 12 peaks in the 720 nm to 760 nm wavelength region.

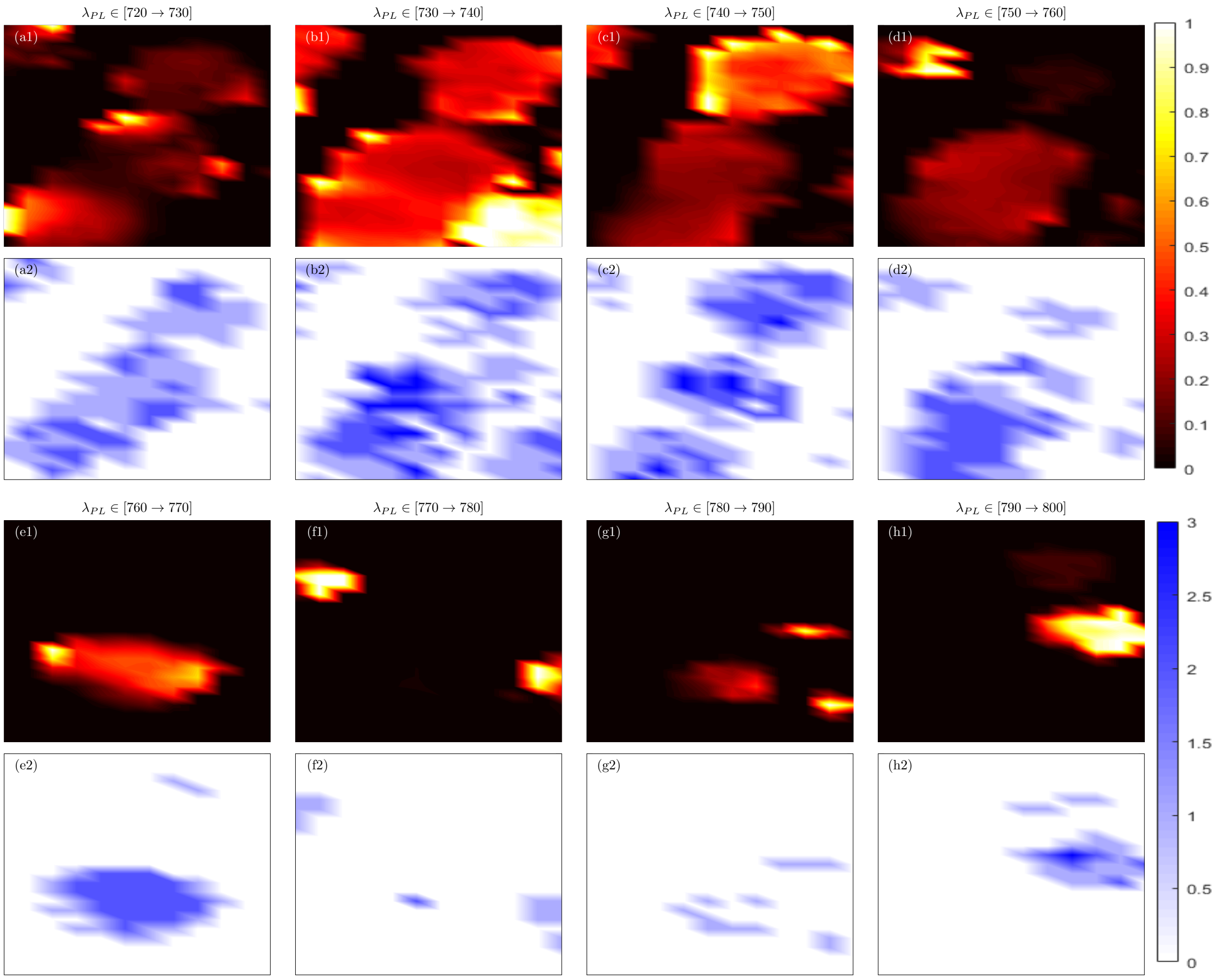

Supplementary Figure 3. Hyperspectral scan. (a1-h1) Normalized total spectral count and **(a2-h2)** number of peaks in the spectral band 720 to 800 nm. Each subplot contains info on a spectral subband of 10 nm wide.

Supplementary Note 5: Second order correlation measurements

Supplementary Figure 4(a) shows the spectrum of spot S5 for wavelengths above 750 nm (below 1.65 eV). Based on this spectrum we assess that the peak of interest (at 1.64 eV) contributes a fraction of about $\rho = 0.76$ (blue shaded area) to the total signal while the rest is due to uncorrelated background (gray shaded areas). The raw normalized coincidence counts are shown in Supplementary Figure 4(b), with a minimum value of 0.43. A fully unconstrained fit (in which we don't require that the minimum of the curve should equal 0.43) however yields $g^{(2)}(0) = 0.69$ and $\tau_{\mathrm{f}} = 7.98$ ns. While the emission exhibits anti-bunching, the $g^{(2)}(0)$ value should drop below 0.5 as a clear sign of single photon emission. When applying background correction (BC) to the raw data, we obtain the red data shown in Supplementary Figure 4(c). For improved visualization, an M-point running average ($M = 11$) was applied to reduce the noise (green data). These data are shown in the main text of the paper. The running average $g^{(2)}_{\mathrm{RA}}(\tau)$ data at each time τ are obtained using the formula

$$g^{(2)}_{\mathrm{RA}}(\tau) = \frac{1}{M} \sum_{n=-\frac{(M-1)}{2}}^{\frac{M-1}{2}} g^{(2)}(\tau + nT), \tag{1}$$

with T the timing resolution of the measurement. For the background-corrected data, $g^{(2)}(0) = 0.47$, confirming single photon emission.

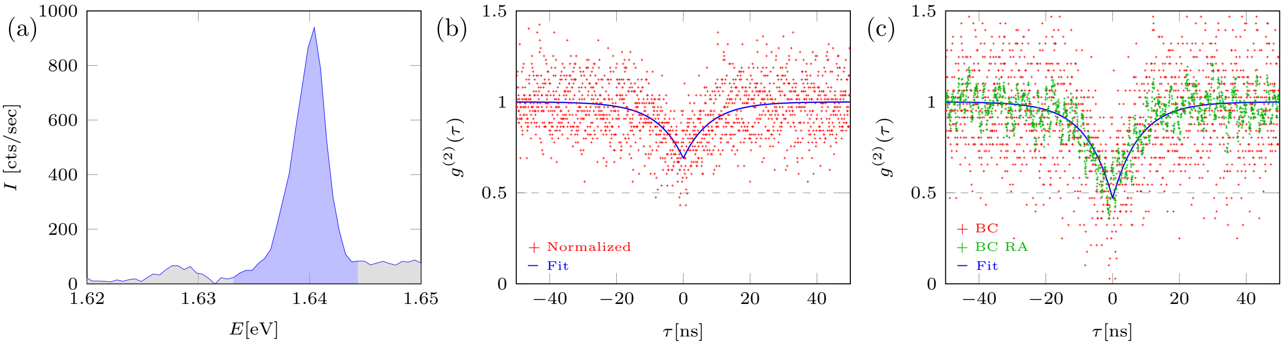

Supplementary Figure 4. Second order correlation measurements. **(a)** Assessment of the signal-to-background ratio ρ (blue=signal, gray=background), based on the spectral count rate of emission with a wavelength above 750 nm. **(b)** Normalized coincidence counts (red) and fit to the normalized, uncorrected data. **(c)** Background-corrected BC (red) and background-corrected running average BC RA (green) coincidence counts with corresponding fit (blue).

Supplementary Note 6: Brightness of the integrated single photon source

The counts incident on SPD1 (which are used to assess the brightness), originate from a light field $\mathbf{E} = E_0(\cos\beta\mathbf{e}_X + \sin\beta\mathbf{e}_Y)$ that consecutively passes through a waveplate with orientation α and a polarizing beamsplitter PBS (for a definition of angles α and β, see Figure 3(d) of the main text). We first express this light field in the frame of the waveplate, which has basis vectors

$$\mathbf{e}_{X'} = \sin\alpha\mathbf{e}_X - \cos\alpha\mathbf{e}_Y \tag{2}$$

$$\mathbf{e}_{Y'} = \cos\alpha\mathbf{e}_X + \sin\alpha\mathbf{e}_Y, \tag{3}$$

such that

$$\mathbf{E} = E_0(\sin(\alpha-\beta)\mathbf{e}_{X'} + \cos(\alpha-\beta)\mathbf{e}_{Y'}). \tag{4}$$

In the frame of the wave-plate, the slow axis ($\mathbf{e}_{Y'}$) obtains a π phase shift, such that the field after the wave-plate and back in the original frame ($\mathbf{e}_X, \mathbf{e}_Y$) is given by

$$\mathbf{E}_{\mathrm{aw}} = -E_0(\cos(2\alpha-\beta)\mathbf{e}_X + \sin(2\alpha-\beta)\mathbf{e}_Y). \tag{5}$$

The PBS does not provide perfect filtering between the $\mathbf{e}_X$ and $\mathbf{e}_Y$ polarization, so we attribute a power transmission of T_X and T_Y to the respective components. So the intensity reaching SPD1 is eventually given by

$$I_{\mathrm{SPD}} = I_0(T_X\cos^2(2\alpha-\beta) + T_Y\sin^2(2\alpha-\beta)), \tag{6}$$

with I_0 the intensity of the original beam. A fit of the SPD1 signal counts as a function of α yields the following fitting values: $T_X = 0.17$, $T_Y = 0.51$ and $\beta_{\mathrm{d}} = 3.9^\circ$ for the dipole emitter and $\beta_{\mathrm{e}} = 18^\circ$ for the excitation polarization respectively. The deviation from the optimal excitation efficiency, i.e. $\beta_{\mathrm{d}} = \beta_{\mathrm{e}}$, is hence only $1 - \cos(\beta_{\mathrm{d}} - \beta_{\mathrm{e}}) \approx 3\%$. So both polarizations are well aligned for this particular emitter, and a negligible increase of $1/\cos(\beta_{\mathrm{d}} - \beta_{\mathrm{e}}) \approx 1.03$ would be expected in the count rate if both polarizations were perfectly aligned.

We moreover approximate the overall transmission loss due to all optics between the half-wave plate and the collection objective to be about $T_{\mathrm{o}} = 50\%$. As discussed in the main text, about $\eta_{\mathrm{NA}} \approx 7\%$ of the total radiation is captured by the collection objective. The maximum SPD1 count rate $I_{\mathrm{SPD}}^{\mathrm{max}} \approx$ 29000 cts/sec was obtained for $\alpha \approx 50^\circ$ and $P_{\mathrm{e}} \approx P_{\mathrm{s}}$. This implies $I_0 \approx 50$ kHz and $I_{\mathrm{s}} = 2I_0 \approx 100$ kHz. Taking into account the remaining transmission and collection losses, the estimated brightness of the single photon source is about $I_0/(T_{\mathrm{o}}\eta_{\mathrm{NA}}) \approx 1.6$ MHz ($I_{\mathrm{s}} \approx 3$ MHz). For an ideal dipole orientation and position, about 7% couples into the forward propagating waveguide mode, which leads to an estimated maximum waveguide-coupled count rate of 100 kHz.

Supplementary Note 7: Evanescently coupled cavity-emitter systems

Master equation

We will describe the evanescently coupled dielectric cavity-emitter system by the same master equation as reported in our earlier work. [1, 2] In a frame rotating at the emitter frequency ω_e, the density matrix ρ satisfies

$$\frac{d\rho}{dt} = -i\Omega[pS_+ + p^\dagger S_-, \rho] + \frac{\gamma_\mathrm{p}}{2}\left(2p\rho p^\dagger - p^\dagger p\rho - \rho p^\dagger p\right) + \frac{\gamma_\mathrm{e}}{2}\left(2S_-\rho S_+ - S_+S_-\rho - \rho S_+S_-\right) + \frac{\gamma^*}{2}\left(2S_z\rho S_z - S_zS_z\rho - \rho S_zS_z\right), \quad (7)$$

with the assumption that the cavity (described by the annihilation operator p) is resonant with the emitter (i.e. $\omega_\mathrm{c} = \omega\mathrm{e}$). The spin operators for the emitter satisfy $S_+ = |e\rangle\langle g|$, $S_- = |g\rangle\langle e|$ and $S_z = \frac{1}{2}\left(|e\rangle\langle e| - |g\rangle\langle g|\right)$. The decay rates γ_p, γ_e and γ^* respectively represent the overall decay rate of the cavity (both due to intrinsic losses and decay into the waveguide), the decay rate of the emitter into the non-guided modes and the dephasing rate of the quantum emitter. The cavity-emitter coupling strength Ω is given by

$$\Omega = \sqrt{\frac{\omega_\mathrm{c}}{2\hbar\epsilon_0}}|\mathbf{p}_\mathrm{d}|\cos\theta_\mathrm{d}\left(\frac{1}{\sqrt{V_\mathrm{c}}}\right) \quad (8)$$

with V_c the cavity mode volume, $|\mathbf{p}_\mathrm{d}|$ the strength of the dipole moment of the emitter and $\theta_\mathrm{d} = \arccos(\mathbf{e}_\mathrm{d}\cdot\mathbf{e}_\mathrm{c})$ the angle between the unit polarization vector of the emitter $\mathbf{e}_\mathrm{d}$ and the cavity field $\mathbf{e}_\mathrm{c}$. [3] The mode volume is defined as

$$V_\mathrm{c} = \frac{\iiint d\mathbf{r}\epsilon(\mathbf{r})|\mathbf{E}_\mathrm{m}(\mathbf{r})|^2}{\epsilon(\mathbf{r}_\mathrm{d})|\mathbf{E}_\mathrm{m}(\mathbf{r}_\mathrm{d})|^2}, \quad (9)$$

with $\mathbf{E}_\mathrm{m}(\mathbf{r})$ the cavity mode field and $\epsilon(\mathbf{r})$ the relative permittivity of the medium. The mode volume is normalized using the mode field at the position of the dipole emitter $\mathbf{r}_\mathrm{d}$ (and hence not using the maximum of the mode field). The strength of the dipole moment $|\mathbf{p}_\mathrm{d}|$ can be related to the emitter decay rate Γ in a uniform dielectric with refractive index n_d through

$$\Gamma = \frac{n_\mathrm{d}\omega_\mathrm{e}^3|\mathbf{p}_\mathrm{d}|^2}{3\pi\epsilon_0\hbar c^3} \Rightarrow |\mathbf{p}_\mathrm{d}| = \sqrt{\frac{3\pi\epsilon_0\hbar c^3\Gamma}{n_\mathrm{d}\omega_\mathrm{e}^3}} \quad (10)$$

such that

$$\Omega^2 = \frac{3\pi c^3}{2n_\mathrm{d}\omega_c^2}\cos^2\theta_\mathrm{d}\left(\frac{\Gamma}{V_\mathrm{c}}\right). \quad (11)$$

The decay rate $\gamma_\mathrm{p} = \gamma_\mathrm{c} + \kappa$ consists of the intrinsic decay rate of the cavity $\gamma_\mathrm{c} = \frac{\omega_\mathrm{c}}{2Q_\mathrm{i}}$ (determined by the intrinsic quality factor Q_i which includes absorption and radiation losses to non-guided modes) and the coupling rate to the guided modes κ. We assume that $\kappa = \chi\gamma_\mathrm{c} = \frac{\omega_\mathrm{c}}{2Q_\kappa}$, such that the loaded quality factor Q of the cavity is

$$Q = \left(\frac{1}{Q_\mathrm{i}} + \frac{1}{Q_\kappa}\right)^{-1} = \frac{Q_\mathrm{i}}{1+\chi}. \quad (12)$$

Single photon extraction efficiency and indistinguishability

To determine the single photon extraction efficiency we assume that the emitter is initialized in the excited state with no photons present in the cavity. The problem can then be described in a basis consisting of just 3 states: $\{|1\rangle = |g,0\rangle, |2\rangle = |g,1\rangle, |3\rangle = |e,0\rangle\}$, respectively corresponding to a state where the emitter is in the ground state and no photons are in the cavity, a state where the emitter is in the ground state and 1 photon is present in the cavity and a state where the emitter is in the excited state and no photon present in the cavity. The rate equations are the same as reported in earlier work. [1, 2, 4] In the basis $\{|1\rangle = |g,0\rangle, |2\rangle = |g,1\rangle, |3\rangle = |e,0\rangle\}$ we get

$$\frac{d\rho_{22}}{dt} = -2\Omega\Im(\rho_{23}) - \gamma_{\mathrm{p}}\rho_{22} \tag{13}$$

$$\frac{d\rho_{33}}{dt} = 2\Omega\Im(\rho_{23}) - \gamma_{\mathrm{e}}\rho_{33} \tag{14}$$

$$\frac{d\Im(\rho_{23})}{dt} = -\frac{\gamma_{\mathrm{p}} + \gamma_{\mathrm{e}} + \gamma^*}{2}\Im(\rho_{23}) + \Omega\rho_{22} - \Omega\rho_{33} \tag{15}$$

and we assume the system is initially in the excited state, i.e. $\rho(t=0) = |3\rangle\langle 3|$. For a system at low temperature (4K) we can safely assume $\gamma^* \ll \gamma_{\mathrm{e}} + \gamma_{\mathrm{p}}$ such that the γ^* can be neglected in the equation for $\Im(\rho_{23})$. This is justified as a typical γ^* at low temperature would be on the order of 10 to 100 GHz [5], while the total cavity decay rate $\gamma_{\mathrm{p}} = \omega_{\mathrm{c}}/(2Q)$ would typically be 1000 GHz for a loaded $Q = 1000$ (at $\lambda = 750$ nm). After solving for $\rho_{22}(t)$, the single photon generation efficiency into the waveguide mode is

$$\eta = \kappa\int_0^\infty dt\rho_{22}(t) = \frac{\kappa}{(\gamma_{\mathrm{e}} + \gamma_{\mathrm{p}})\left(1 + \frac{\gamma_{\mathrm{e}}\gamma_{\mathrm{p}}}{4\Omega^2}\right)}, \tag{16}$$

which is the same equation as obtained before. [1, 2] If γ^* can not be neglected, the system can still be solved analytically but the formula becomes quite cumbersome. One could then resort to a full numerical approach as well. The formula for the indistinguishability of photons coupled into the guided mode, derived by Grange et al.[4], depends on the regime within which the system falls:

- coherent coupling regime: $2\Omega > \gamma_{\mathrm{p}} + \gamma_{\mathrm{e}} + \gamma^*$:

$$V = \frac{(\gamma_{\mathrm{e}} + \gamma_{\mathrm{p}})(\gamma_{\mathrm{e}} + \gamma_{\mathrm{p}} + \gamma^*/2)}{(\gamma_{\mathrm{e}} + \gamma_{\mathrm{p}} + \gamma^*)^2} \tag{17}$$

- incoherent coupling regime: $2\Omega < \gamma_{\mathrm{p}} + \gamma_{\mathrm{e}} + \gamma^*$:
  - Bad cavity limit: $\gamma_{\mathrm{p}} > \gamma_{\mathrm{e}} + \gamma^*$:

$$V = \frac{\gamma_{\mathrm{e}} + R}{\gamma_{\mathrm{e}} + R + \gamma^*} \tag{18}$$

  - Good cavity limit: $\gamma_{\mathrm{p}} < \gamma_{\mathrm{e}} + \gamma^*$:

$$V = \frac{\gamma_{\mathrm{e}} + \frac{\gamma_{\mathrm{p}}R}{\gamma_{\mathrm{p}}+R}}{\gamma_{\mathrm{e}} + \gamma_{\mathrm{p}} + R} \tag{19}$$

with

$$R = \frac{4\Omega^2}{\gamma_{\mathrm{e}} + \gamma_{\mathrm{p}} + \gamma^*}. \tag{20}$$

The above formulas are used to calculate η and ηV as a function of mode volume and $\kappa/\gamma_{\mathrm{c}}$ in the main text of the paper.

Optimum single photon extraction

Substituting $\kappa = \chi\gamma_c$ into the formula for single photon extraction yields

$$\eta = \left(\frac{\chi}{1+\chi+\frac{\gamma_e}{\gamma_c}}\right)\left(\frac{1}{1+\frac{\gamma_e\gamma_c}{4\Omega^2}(1+\chi)}\right), \tag{21}$$

Solving for $d\eta/d\chi = 0$ yields the optimum value for χ to maximize η for a given $\Omega^2 \propto 1/V_c$. The optimum reads

$$\chi_{\text{opt}} = \sqrt{\left(1+\frac{\gamma_e}{\gamma_c}\right)\left(1+\frac{4\Omega^2}{\gamma_e\gamma_c}\right)} = \frac{\kappa_{\text{opt}}}{\gamma_c}. \tag{22}$$

Comparison with metallic nanostructures

A: Metallic nanowire

The coupling of single emitters to metallic nanowires has been studied analytically in Supplementary Reference [6]. The coupling efficiency to the surface plasmon mode can be obtained through $\beta = \frac{P}{1+P}$ with $P = \frac{\Gamma_{\text{pl}}}{\Gamma_{\text{rad}}+\Gamma_{\text{nrad}}}$ and Γ_{pl} the decay rate to the plasmon mode, Γ_{rad} the radiative decay rate to free space modes and Γ_{nrad} the decay rate to non-radiative modes respectively. In Supplementary Reference [6] it is shown that P can easily be 100, depending on nanowire radius and distance between the emitter and the metal surface, implying that β can reach unity in such structures. However, this result holds when the dipole polarization of the emitter is oriented normal to the nanowire surface. When the emitter polarization is oriented tangential to the nanowire, it will not couple to the surface plasmon mode. Usually, the dipole polarization of 2D-based emitters is oriented in the plane of the material, so when the material is transferred on the nanowire, it is unlikely the dipole will couple efficiently to the surface plasmon mode. Hence, we deem this is not the most convenient geometry to enhance funneling into a guided mode. Moreover, the non-radiative decay rate becomes extremely large when the emitter approaches the metal surface [6], so the wire needs to be coated with a capping layer in any case to avoid that the emission gets totally quenched.

B: Metal-insulator-metal waveguide

In order to assess the coupling strength between a quantum emitter and a metal-insulator-metal (MIM) waveguide, we simulate a structure as shown in Supplementary Figure 5(a). It consists of a Au-SiO_2-Au MIM structure, with a gap of 100 nm and height of 250 nm. Assuming the 2D material is put on top of the structure, we simulate the coupling efficiency β to the guided mode (both forward and backward) of a dipole emitter placed at $y = 125$ nm (top of the structure) and with varying x_d. Supplementary Figure 5(b) shows β for varying positions of the dipole. It is clear that β remains fairly constant and equals 20%. By decreasing the gap width to 50nm, one can achieve $\beta = 44.41\%$ for this particular structure. Supplementary Figure 5(c) shows β as a function of the angle α between the polarization direction and the $z-$axis (propagation direction). When the dipole orientation aligns with the propagation direction, the coupling to the guided mode vanishes. While one can achieve much higher coupling efficiencies with this structure as compared to the coupling with a normal waveguide, it should also be noted that the plasmon mode needs to be converted to a regular dielectric waveguide mode for propagation over larger distances because the plasmon mode is extremely lossy. For the structure in Supplementary Figure 5(a) the loss amounts 11401 dB/cm, i.e. propagation length of 3.81μm versus a propagation length on the order of 1cm for a dielectric SiN waveguide. So one could envision a platform with a short MIM-section that adiabatically tapers into a dielectric waveguide to enhance the overall collection but this adds considerable fabrication challenges. The use of dielectric cavities moreover also allows near-unity coupling efficiencies and can be fully processed in the SiN platform itself, so there is no direct benefit of using a metal-based structure.

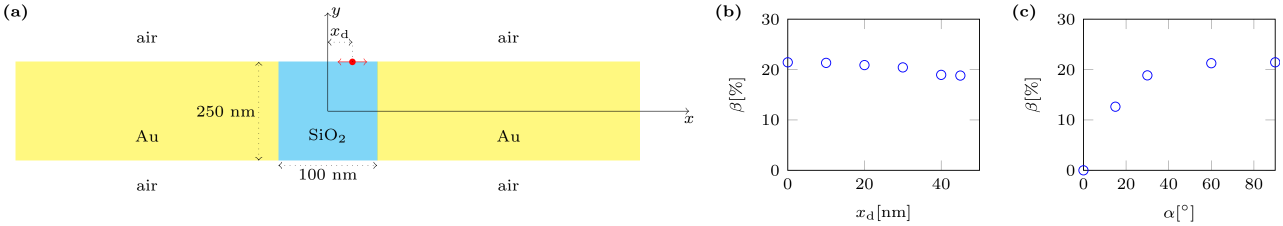

Supplementary Figure 5. Metal-insulator-metal (MIM) waveguide. (a) Schematic of MIM waveguide. The waveguide is made of gold metal pads that extend infinitely in both x and z, with a height of 250 nm in the y direction. The structure is air-cladded and has a gap width of 100 nm, filled with SiO_2 ($n = 1.45$). For the refractive index of Au, we used the built-in Johnson and Christy model in Lumerical FDTD Solutions. A dipole emitter is placed on top of the structure at a position x_d with respect to the center of the gap. **(b)** Coupling efficiency to the plasmon mode (β) as a function of x_d. **(c)** Coupling efficiency to the plasmon mode (β) for a structure with $x_\mathrm{d} = 0$ nm as a function of the angle between the polarization direction and the $z-$axis (propagation direction).

Supplementary References

[1] Peyskens, F., Chang, D., Englund, D. Integrated nanoplasmonic quantum interfaces for room-temperature single-photon sources. *Phys. Rev. B* **96**, 235151 (2017).

[2] Peyskens, F., Englund, D. Quantum photonics model for nonclassical light generation using integrated nanoplasmonic cavity-emitter systems. *Phys. Rev. A* **97**, 063844 (2018).

[3] Steck, D.A. *Quantum and Atom Optics.* Available online at `http://steck.us/teaching` (revision 0.12.5, 26 January 2019).

[4] Grange, T., Hornecker, G., Hunger, D., Poizat, J.-P., Gérard, J.-M., Senellart, P., Auffèves, A. Cavity-Funneled Generation of Indistinguishable Single Photons from Strongly Dissipative Quantum Emitters. *Phys. Rev. Lett.* **114**, 193601 (2015).

[5] Luo, Y., Shepard, G.D., Ardelean, J.V., Rhodes, D.A., Kim, B., Barmak, K., Hone, J.C., Strauf, S. Deterministic coupling of site controlled quantum emitters in monolayer WSe2 to plasmonic nanocavities. *Nat. Nanotech.* **13**, 1137–1142 (2018).

[6] Chang, D.E., Sørensen, A.S., Hemmer, P.R., Lukin, M.D. Strong coupling of single emitters to surface plasmons. *Phys. Rev. B* **76**, 035420 (2007).
